# A Modular Hybrid Framework for CEST MRI Development and Clinical Translation Using pTx‐Pulseq Preparation With Open‐ and Closed‐Source Readouts and ONNX Mapping

**DOI:** 10.1002/mrm.70596

**Published:** 2026-09-24

**Authors:** Martin Freudensprung, Junaid R. Rajput, Patrick Liebig, Simon Weinmüller, Ludwig Singer, Nikita Vladimirov, Or Perlman, Armin Michael Nagel, Arnd Doerfler, Moritz Zaiss

**Affiliations:** ^1^ Institute of Neuroradiology Uniklinikum Erlangen, Friedrich‐Alexander‐Universität Erlangen‐Nürnberg Erlangen Germany; ^2^ Siemens Healthcare GmbH Erlangen Germany; ^3^ Pattern Recognition Lab Friedrich‐Alexander‐University Erlangen‐Nürnberg Erlangen Germany; ^4^ School of Biomedical Engineering Tel Aviv University Tel Aviv Israel; ^5^ Sagol School of Neuroscience Tel Aviv University Tel Aviv Israel; ^6^ Institute of Radiology Uniklinikum Erlangen, Friedrich‐Alexander‐Universität Erlangen‐Nürnberg (FAU) Erlangen Germany; ^7^ Medical Physics in Radiology German Cancer Research Center (DKFZ) Heidelberg Germany; ^8^ Department Artificial Intelligence in Biomedical Engineering (AIBE) Friedrich‐Alexander‐Universität Erlangen‐Nürnberg Erlangen Germany

**Keywords:** CEST, MRF, ONNX, pTx, Pulseq, UHF‐MRI

## Abstract

**Purpose:**

Chemical exchange saturation transfer (CEST) MRI offers unique metabolic insights for imaging of cancer, stroke, or neurodegeneration. However, the rapid expansion of CEST preparation schemes and evaluation techniques creates a bottleneck; novel methods often remain complex prototypes requiring offline processing, preventing clinical adoption.

**Methods:**

To bridge this gap, we propose a hybrid sequence and reconstruction framework. This approach combines a flexible, pTx‐enabled preparation block using the open‐source Pulseq standard with a closed‐source snapshot readout, and an interchangeable ONNX‐based neural network for online post‐processing.

**Results:**

We validated this framework at 7 T using three distinct applications: deepWASABI for B0 and B1 prediction, a protein CEST approach for B1‐corrected CEST mapping, and semi‐solid MT MRF for quantifying magnetization transfer rates and semi‐solid proton volume fractions. By utilizing closed‐source readouts, we leverage advanced proprietary image reconstructions essential for high‐resolution whole‐brain coverage. Simultaneously, the integrated ONNX network processes contrast‐weighted images directly at the scanner to generate parameter maps online.

**Conclusion:**

This hybrid architecture eliminates complex compilation steps, allowing deployment by simply copying Pulseq and ONNX files. By enabling push‐button execution and immediate clinical data storage, this platform significantly accelerates the development, evaluation, and dissemination of advanced CEST MRI methods and forms a blueprint for scaling any magnetization‐prepared MRI approach at any system.

## 1 Introduction

Chemical exchange saturation transfer (CEST) MRI is a metabolic MRI technique [1] with the ability of tumor detection and subtyping [2], and novel insights into stroke or neurodegeneration [3, 4, 5]. The CEST field has a growing number of new CEST preparation schemes as well as new evaluation techniques and tailored neural networks [6, 7, 8, 9]. Both the development of evaluations and new CEST MRI sequences are challenging and time‐consuming, as novel methods often exist solely as prototypes. They can only be run by experts. However, an integration into larger clinical studies requires robust push‐button solutions that can be run by general MR operators. Even if the prototype sequence can be simply executed, the post‐processing procedures of novel methods often run only offline. Therefore, the integration of new methods into clinical studies is hindered or delayed. Furthermore, methodological developments in CEST MRI typically focus on preparation and evaluation, while the readout is often adopted from established standard sequences.

To address these challenges, we propose a hybrid sequence, combined with a reconstruction framework. The sequence is based on (i) a preparation block defined in the flexible open‐source Pulseq standard [10, 11], which we extended for pTx abilities [12] and (ii) a closed‐source readout sequence. In our case, it is a snapshot gradient echo (GRE) sequence [13], including advanced image reconstruction. The reconstruction section is (iii) an interchangeable ONNX‐based (Open Neural Network Exchange, https://github.com/onnx) neural network post‐processing tool for parameter map estimation and DICOM image generation.

While a pure Pulseq implementation is possible, the proposed approach allows to have all benefits of the closed‐source readout part, including all sequence options, as well as online image reconstruction tools. All advanced closed‐source deep learning reconstructions of the vendor or third‐parties can be used, which are essential to achieve high resolution and whole brain coverage herein. The resulting magnitude images can be further processed and analyzed using the corresponding, interchangeable ONNX network.

This hybrid approach enables the new CEST method to be deployed to the scanner without requiring the sequence to be recompiled. It is only necessary to transfer the seq‐file for preparation and the ONNX files for reconstruction to the scanner. The proposed approach significantly accelerates method development—including the critical clinical evaluation phase—by making the sequencing and reconstruction pipeline available through exchange platforms, currently for one vendor, with others to follow.

Moreover, this hybrid sequence forms an attractive prototyping platform, as many of recent developments, open source or closed source, can be combined, which is important also for realistic judgment of performance and hypothesis generation in preliminary tests of newest ideas.

The core contribution of this work is the coherent integration of these components into a single, scanner‐deployable framework. Beyond this integration, several individual components were newly developed or substantially extended. We advanced our pTx extension for Pulseq toward the hybrid approach presented here and additionally realized a pure Pulseq version including preparation and 2D readout, complementing the closed‐source readout path. The ONNX‐based inference, first presented in preliminary form [14], was generalized from a fixed normalization and B1‐mapping scheme into an interchangeable format that allows networks to be exchanged without developing a new vendor sequence, including the online B1 correction required at ultra‐high field.

We tested our full hybrid pipeline for three different CEST‐based methods. First, we introduce a deepWASABI approach, which predicts B_1_ and B_0_ maps using a neural network. Secondly, the PICAE [15] approach predicts CEST maps based on 54 offsets plus integrated B_1_ mapping. And finally, a CEST MR fingerprinting (MRF) method yields semisolid magnetization transfer (ssMT) proton volume fraction and exchange rate maps [16, 17]. In all cases, the pulses can have arbitrary pTx RF shims, and a neural network tailored to the respective data generates the predicted different (CEST) contrasts directly at the scanner. Thus, it enables direct transfer to clinical image storage systems.

## 2 Methods

Our approach is based on the further development of the approach by Herz et al. [10]. The sequence interpreter introduced here is a hybrid GRE sequence developed in the Siemens IDEA framework, which allows CEST preparation in the Pulseq standard (Figure 1A,B) and using a closed‐source readout (Figure 1C,D). This has the advantage that almost all preparation pulses to be used, even beyond classic CEST preparations, can be combined with closed‐source readouts for image acquisition. This sequence uses a Pulseq version that enables RF pulse‐specific RF shimming [12]. When the Pulseq preparation is selected, the sequence timing is controlled by the Pulseq file. The user can select a Pulseq file from a drop‐down menu. This Pulseq file differs from a classic Pulseq file only in the definition of the ADC. Instead of a readout pattern defined within Pulseq, this file requires only a single ADC event, a so‐called pseudo‐ADC. This event is detected by the sequence interpreter and triggers a snapshot GRE readout. The Pulseq preparation can also be deselected, in which case only a single 3D readout is performed. The online evaluation follows the same principle. When it is activated via a checkbox, any neural network stored as ONNX File in the corresponding folder can be selected to post‐process the acquired data (Figure 1E,F).

**FIGURE 1:**
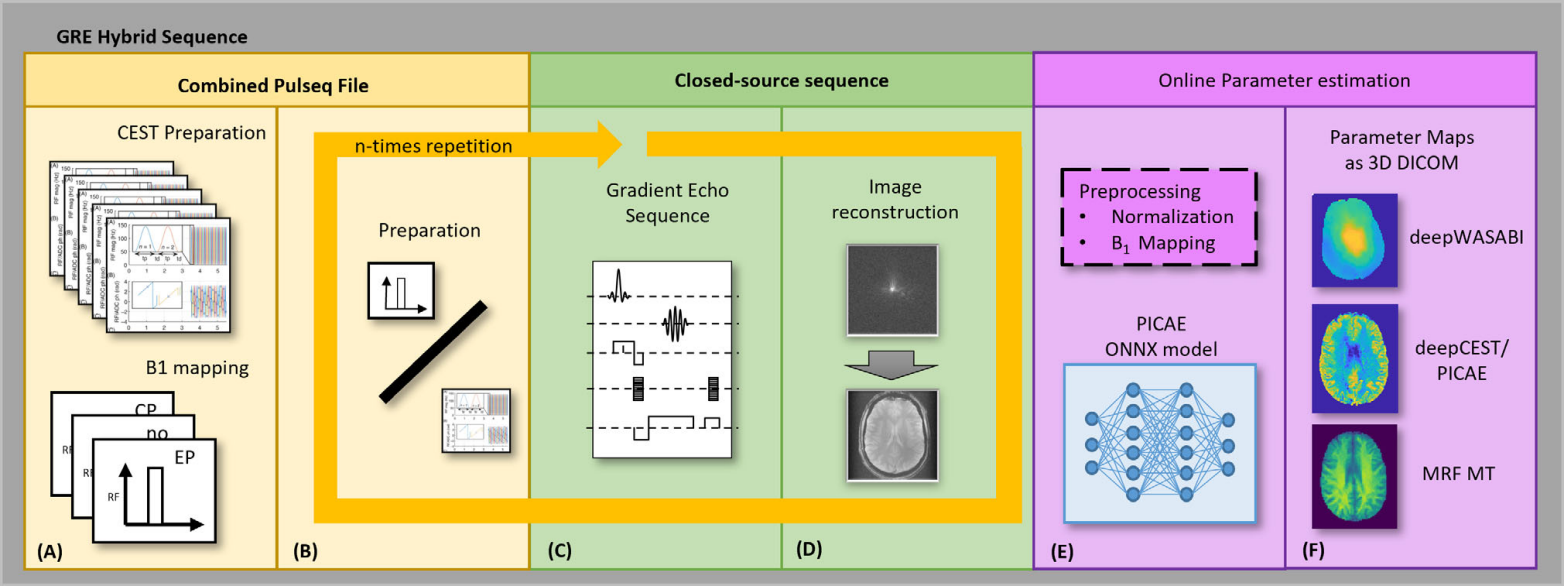
Scheme of the hybrid sequence. An advanced user creates a new Pulseq‐file including preparation pulses for B_1_ Mapping and CEST (A). An ADC Event in the Pulseq File triggers n‐times a Gradient Echo Sequence (B, C). The reconstruction of every repetition is also done by the closed‐source sequence (D). The reconstructed 3D volumes are now further processed within the reconstruction pipeline (E). If necessary, the data can be normalized or a B_1_^+^ map can be generated. The 3D volumes are loaded pixel by pixel into the ONNX model. The calculated maps are scaled to the DICOM format and made available to the user directly with the generated offset DICOMs (F).

### 2.1 Framework Architecture

The following sections describe the building blocks of the framework: the pTx‐enabled Pulseq preparation, the closed‐ and open‐source readouts, the ONNX‐based parameter map estimation, and the standardized CEST preparation.

#### 2.1.1 Pulse‐Specific RF Shimming for Pulseq

Ultra‐high field MRI scanners have a more inhomogeneous B_1_^+^ field [18, 19]. To address this problem, we extended the Pulseq standard to individually control the magnitude and phase of each transmit channel in a coil array. Here, we used an 8Tx/32Rx head coil (Nova Medical, USA) with 8 transmit coils. The benefits of using different shim modes for CEST preparation have already been addressed with the MIMOSA approach by Liebert et al. [20], which alternates between one pulse in circular polarization (CP) mode and one in elliptical polarization (EP) mode. With our Pulseq extension [12], we enable the control of each individual RF pulse to be modulated individually to mitigate inhomogeneities in the B_1_^+^ field. To further evaluate this capability, we applied the PUSHUP approach [21] and measured the resulting B1+ field distributions for two distinct shim configurations: a whole‐brain optimized shim and a cerebellum‐optimized shim. The corresponding B1+ maps are shown in Figure S2, demonstrating that our extension allows other RF shimming approaches to be readily incorporated into Pulseq‐based sequences.

#### 2.1.2 Closed‐Source Readouts

##### 2.1.2.1 Conventional Snapshot GRE Sequence and Reconstruction

The conventional 3D snapshot GRE sequence [10, 13] consisted of CEST preparation blocks and a slab‐selective 3D RF and gradient‐spoiled gradient echo (GRE) readout with Cartesian‐centric rectangular spiral reordering. To reduce the number of k‐space lines, we omit the corners of the k‐space (elliptical scanning). The first aim is to extend the standard snapshot GRE sequence from a slab‐selective readout to cover a whole brain. Using an acceleration factor of GRAPPA 6 [22] (no undersampling artifacts visible), combined with a nominal resolution of 2x2 mm and a slice thickness of 4 mm, we reach a whole brain coverage in one shot. This sequence we termed conventional GRE Hybrid (cGRE). The sequence parameters can be found in Table 1. The reconstruction for the cGRE sequence is a conventional GRAPPA reconstruction. Only magnitude images are generated for further evaluation. This can be requested via the Siemens C2P Platform.

**TABLE 1:** Protocol parameters of the cGRE sequence provided on the C2P platform and the version supported by deep learning reconstruction in the work in progress CEST package (dlGRE).

Protocol	Whole brain cGRE	Whole brain dlGRE	High resolution dlGRE
Resolution [mm^3^]	2.00 × 2.00 × 4.00	2.00 × 2.00 × 4.00	1.00 × 1.00 × 2.00
Interpolated	(1.00 × 1.00 × 4.00)	(1.00 × 1.00 × 4.00)	(0.50 × 0.50 × 1.00)
FOV [mm^3^]	224 × 180 × 144	224 × 180 × 144	224 × 180 × 140
Acceleration	GRAPPA 6	CAIPIRINIA 6	CAIPIRINIA 12
Lines per Shot	526	432	744
TR[ms]/TE[ms]/FA[°]	4.1/1.91/6	4.10/1.67/6	4.10/1.91/6

##### 2.1.2.2 Deep‐Learning CEST Sequence and Reconstruction

The extended Pulseq pTx functionality for preparation pulses, as well as the reduced version for the PICAE network of the ONNX Online CEST Map estimation is implemented in a highly‐accelerated snapshot readout with deep learning reconstruction capabilities. This sequence (dlGRE) is based on a VIBE [23] sequence, allowing deep learning‐based reconstruction techniques [24] that use data consistency and denoising within each iteration step and is based upon the variational network approach [25]. This enables higher acceleration factors for GRAPPA [22] or CAIPIRINIA [26] up to a factor of 12 without introducing substantial image artifacts. The vendor's sequence prototype is capable of accessing the B_0_ and B_1_ maps acquired during the preparation scan of the scanner. No further B_1_ mapping is required, which reduces the measurement time.

#### 2.1.3 Open‐Source Readouts

The closed‐source readout described above can be replaced by an open‐source readout using the following function from the Pulseq‐CEST Library [https://github.com/kherz/pulseq-cest-library/tree/master/utils]. This function combines the CEST preparations with a readout implemented in Pulseq, for which an exemplary 2D readout is also provided. If the ADC labels are set correctly as implemented there, the vendor standard reconstruction software can generate the images directly on the scanner. By additionally incorporating the ONNX‐based reconstruction pipeline extension described in the following section, it is now possible to run a fully Pulseq‐based sequence and obtain the neural‐network‐inferred parameter directly on the scanner as well.

#### 2.1.4 Online ONNX Parameter Map Estimation

The ability to calculate parameter maps directly online on the scanner in the reconstruction pipeline is the main new feature. For this purpose, an ONNX network can be selected on the special card of the sequence. Analogous to the Pulseq files, the ONNX networks are stored in the corresponding folder on the scanner and can therefore be exchanged and updated independently of the sequence. The desired network is then selected via a drop‐down menu. The input of the neural network is magnitude images and applied voxelwise. An INI file next to the ONNX file allows additional settings, such as naming the output maps, masking the data, transferring the results in floating point numbers to the integer system of DICOM data, the type of normalization (M_0_, Baseline or no normalization), and optional B_1_ map creation. Samples of ONNX networks with the corresponding Pulseq Sequences files can be downloaded from the Pulseq CEST Library [27].

#### 2.1.5 Offline ONNX Parameter Map Estimation

Correctly generating the network, saving it in ONNX format, and fine‐tuning the scale and bias values for transformation into DICOM format takes time. To avoid having to do this on the scanner, we have created an Offline ONNX Parameter estimation framework in Python (https://gitlab.rrze.fau.de/cest/offline_onnx_parameter_estimation). The functions contained in the toolbox can read measurement data in DICOM format directly from the scanner. These are stored in Niftis by the dcm2nii tool [28]. Normalization and B_1_ mapping are then performed in the same way as the calculation on the scanner. The evaluation can be performed either voxel by voxel or in batches, which significantly speeds up the calculation time. The output is then in the float range and in the scaled DICOM range. An additional function of the toolbox is the ability to re‐scale previously calculated DICOM results from the scanner back to float values using the INI file.

#### 2.1.6 Standardized CEST Preparation Based on the Pulseq‐CEST Library

For the CEST saturation block, parameters according to Schuenke et al. [29] for the deepWASABI experiment, Mennecke et al. [19] and Rajput et al. [15] for the PICAE experiment, and Power et al. [17] (with proper modifications for 7 T) for the MRF experiment were used. The exact definitions of the preparation modules were defined using Pulseq‐CEST (https://pulseq-cest.github.io) and are available open source in the Pulseq‐CEST library [27].

One crusher gradient was applied after each preparation to spoil residual transverse magnetization before the readout. Saturation and readout were repeated at different off‐resonance frequencies, and at −300 ppm for an unsaturated reference image.

### 2.2 Applications

Using this framework, we implemented three CEST‐based applications. DeepWASABI was newly developed in this work and is described in full detail, whereas PICAE and the semisolid MT‐MRF approach are integrated as demonstrators based on prior work [15, 16], with only the adaptations made here described.

#### 2.2.1 DeepWASABI

The WASABI approach introduced by Schuenke et al. [29] is an example to transfer an analytical function of offline CEST calculations into a trained neural network. The WASABI fit function can be described as follows:

(1) $$Z\left(\Delta \omega \right)=\left\|c-d\cdot \sin^{2}\left(\tan^{-1}\left(\frac{\gamma B_{1}}{\Delta \omega -\delta \omega }\right)\right)\cdot \sin^{2}\left(\sqrt{{\left(\gamma B_{1}\right)}^{2}+{\left(\Delta \omega -\delta \omega \right)}^{2}}\cdot \frac{t_{p}}{2}\right)\right\|$$

where (c) and (d) are empirical fit parameters, (γ) denotes the gyromagnetic ratio $1H:\gamma =\frac{\omega_{0}}{B_{0}}=2\pi \cdot 42.578\frac{\;\mathrm{MHz}}{T}$, $\left(B_{1}\right)$ is the transmit RF field amplitude, and (δω) represents the local frequency shift. Furthermore, $\left(t_{p}\right)$ denotes the RF pulse duration and (Δω) the applied radiofrequency offset relative to the Larmor frequency. The term (Δω−δω) therefore describes the effective frequency offset within a voxel [29].

The absolute value of the function was used because the ONNX inference is based on reconstructed magnitude images. Training was performed on a voxel‐wise basis. The training data were generated using a predefined lookup table spanning B_1_ values from 0.5 to 1.5 × B_1rms_, B_0_ offsets from −0.6 to 0.6 ppm, c values from 0.6 to 0.95, and d values from 1.1 to 1.85. Fixed sequence parameters were B_1rms_ = 5.12 μT, T_p_ = 3840 ms, a frequency of 297.18 Hz, and 21 frequency offsets calculated according to the equation above. We tested the performance of the trained network on two WASABI sequences with different B_1_ shims: one in CP mode and one in EP mode. Both sequences, as well as the ONNX network, can be found in the Pulseq CEST Library [27].

As reference we used the standard offline WASABI evaluation approach described by Schünke et al. [29]. This can be found here (https://gitlab.cs.fau.de/al52aros/cest_eval/-/tree/mrm.70596 [30]).

#### 2.2.2 Low Power CEST/PICAE With B_1_ Correction

We integrated the PICAE CEST evaluation of Rajput et al. [15] into the hybrid sequence. This approach uses an unsupervised physics‐informed conditional autoencoder (PICAE) that integrates Lorentzian modeling to perform voxel‐wise B_1_ correction and isolate metabolic maps from CEST MRI data. This network can be stored as an ONNX network. For the preparation, we use a pulse train with 54 offsets, 120 sinc‐gauss pulses with an RF shim alternating MIMOSA mode [20], *t*_p_ = 15 ms, *t*_d_ = 10 ms, *T*_prep_ = 3 s, *B*_1rms_ = 0.95 μT. As already shown here [14], it is possible to integrate the B_1_ map measurement with the NN‐based evaluation into a single measurement. For B_1_ mapping, we use the methodology described below. To normalize the data, two different techniques are possible: solely M_0_ normalization or baseline normalization. For the PICAE preparation, dummy scans are needed because there is a drift at the beginning of the measurement [31]. Therefore, 10 dummy scans are added at the beginning to reach a steady state. The M_0_ scan is a far off‐resonance preparation. The second option is a baseline normalization where a baseline is calculated from the first and last measurements of the CEST spectrum. Each measured 3D image is now normalized with a normalization value adjusted to the baseline. In the proposed hybrid sequence, the normalization method—none, M_0_ scan, or baseline—can be configured in the INI file. The number of dummy scans can also be freely selected.

B_1_ mapping is necessary to correct for strong B_1_^+^ inhomogeneities in 7 T MRI. The use of MIMOSA preparations [20] makes it necessary to calculate the MIMOSA map from the CP and EP. These CP and EP maps can be created using the method developed by Chung, Kim et al. [32] Two images are needed to generate CP or EP B_1_ map. The first one is prepared by a slice‐selective pre‐conditioning (SSPre) RF pulse, the second image has no pre‐conditioning pulse and is the reference (PD). Both with an RF shim alternating MIMOSA mode images uses a fast 3D GRE sequence as readout. They are added after the CEST measurements using the same readout scheme. Here, the preparation module defined in Pulseq are the B_1_ mapping preparations. The B_1_ map is calculated by

(2) $$B_{1}=\cos^{-1}\frac{\text{SSPre}}{\mathrm{PD}}/\alpha^{\mathrm{nom}}$$

where (SSPre) denotes the prepared image, (PD) the reference image and $\alpha^{\mathrm{nom}}$ the nominal flip angle (70°). Having the B1 map for the EP and CP mode, it follows for the MIMOSA B_1_ map.

(3) $$B_{1,\text{MIMOSA}}=\sqrt{\frac{{\mathrm{CP}}^{2}+{\mathrm{EP}}^{2}}{2}}$$

#### 2.2.3 MRF

Inspired by recent saturation transfer magnetic resonance fingerprinting (MRF) studies [17, 33], a semisolid magnetization transfer (MT) MRF acquisition protocol was implemented, generating 23 raw, molecular information encoding images using a spin lock saturation train (13 off‐resonant spin lock pulses, *T*_lock_ = 100 ms, 50% duty‐cycle). The protocol varied the saturation pulse power between 0 and 3.2 μT (average pulse amplitude; the exact saturation pulse train parameters are provided in the provided .seq file) and the frequency offset between 6 and 14 ppm. A simulated dictionary was then generated using the open‐source code available in https://github.com/momentum-laboratory/molecular-mrf accommodating the tissue parameter combinations described by Perlman et al. [33] and relative B_1_ inhomogeneities spanning 0.1–1.3 (relative units, with “1” considered as perfect shimming). Two dictionary variants were created—one with a small amount of noise and one with realistic noise levels (40–46 dB for typical MT‐MRF measurements) [34].

A fully connected neural network with two hidden layers of 300 neurons each was implemented in PyTorch using the code available in ref. [16]. The network was trained using the simulated dictionary to receive a raw MRF trajectory and an explicit B_1_ inhomogeneity value and output the corresponding semisolid MT proton volume fraction (f_ss_) and exchange rate (k_ssw_). For comparison, another network was trained using the exact conditions, yet without B_1_ input.

### 2.3 Experiment Setup

The measurements were applied in healthy volunteers (two female and two male, aged 23 to 42 years). For the reproducibility assessment, four additional healthy volunteers (Sub1, f/24; Sub2, f/23; Sub3, m/23; Sub4, m/27) were measured with the cGRE Sequences. Each was scanned on two different days at the same time of day. Further details on reproducibility are provided in Figure S3. To confirm that the pixel‐wise evaluation is not affected by anatomical anomalies, representative CEST maps from two patients (m45, f35) are provided in Figure S4. All measurements were performed under approval of our local ethics committee. Scanning was performed on a 7 T whole‐body MRI system (MAGNETOM TERRA.X, Siemens Healthcare, Erlangen, Germany) and an 8Tx/32Rx head coil (Nova Medical, USA).

## 3 Results

### 3.1 deepWASABI

The results of WASABI Fit compared to the results of the neural network output (ONNX) show a high degree of consistency overall (Figure 2). The B_1_ map of the CP mode shows less differences compared to the EP mode. Mainly in the center, differences are visible due to signal cancellations. B_0_ maps match for both modes. Differences in B_0_‐maps acquired in EP and CP modes are also very small, which demonstrates robustness against strong B_1_ fluctuations. The regression plots still show outliers in the B_1_ maps, but since all voxels within the brain mask are displayed here, these can be identified as individual outliers. This confirms the high r and R^2^ values. The scatter plots for the B_0_ values show that the values calculated in the NN are all within a range of ±0.2 ppm. Higher values are achieved with a fit. This can be clearly seen in the subplots (N/M) and (R/S) in the nasal areas, where the B_0_ inhomogeneity is particularly high. Overall, it can be seen that the fit results appear to be somewhat more uniform.

**FIGURE 2:**
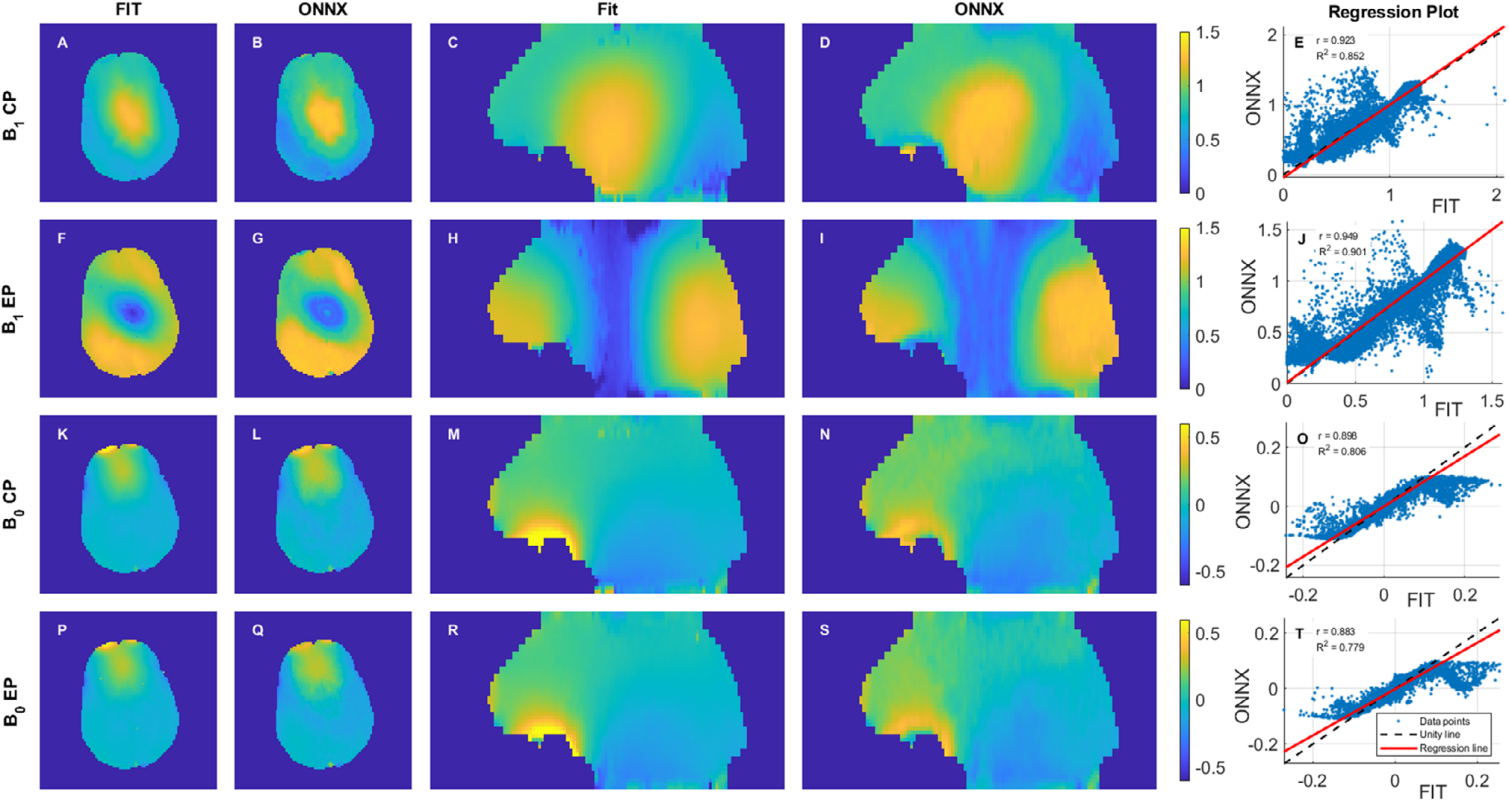
DeepWASABI, The different evaluation results between the fit and the direct output of the neural network (ONNX) in a transverse and a sagittal slice. The plot shows the results of two measurements, one with a CP and one with an EP preparation. The B_1_ and B_0_ maps are shown for each of these two measurements. At the end of each row, a scatter plot is plotted with a voxel‐by‐voxel comparison of all values within the 3D mask between the FIT and the ONNX.

### 3.2 deepCEST—PICAE

The evaluation of the more complex PICAE network also provides convincing results. The neural network returns identical values at the scanner as in an offline implementation. This can be seen very clearly in the regression plot in Figure 3. There, the unit line disappears behind the regression line, and the r and R^2^ values are also above 0.99. The output values at the scanner are scaled to the DICOM format, which is why the images in the first row show less contrast. However, as is usual with classic MRI data, the user at the scanner can select the windowing themselves and thus choose the contrast as required. The near‐perfect agreement between online and offline evaluation serves as proof that skipping dummy scans, normalization using baseline correction, and online B_1_ calculation work. The few outliers visible in the scatter plot are attributed to rounding errors introduced by the conversion of floating‐point values to the integer‐based DICOM format and back. However, the evaluation time for this complex network is slightly longer than for the deepWASABI network but still only takes a few minutes. As a rule, however, it is reconstructed and evaluated by the end of the entire measurement and can be exported together with the anatomical data.

**FIGURE 3:**
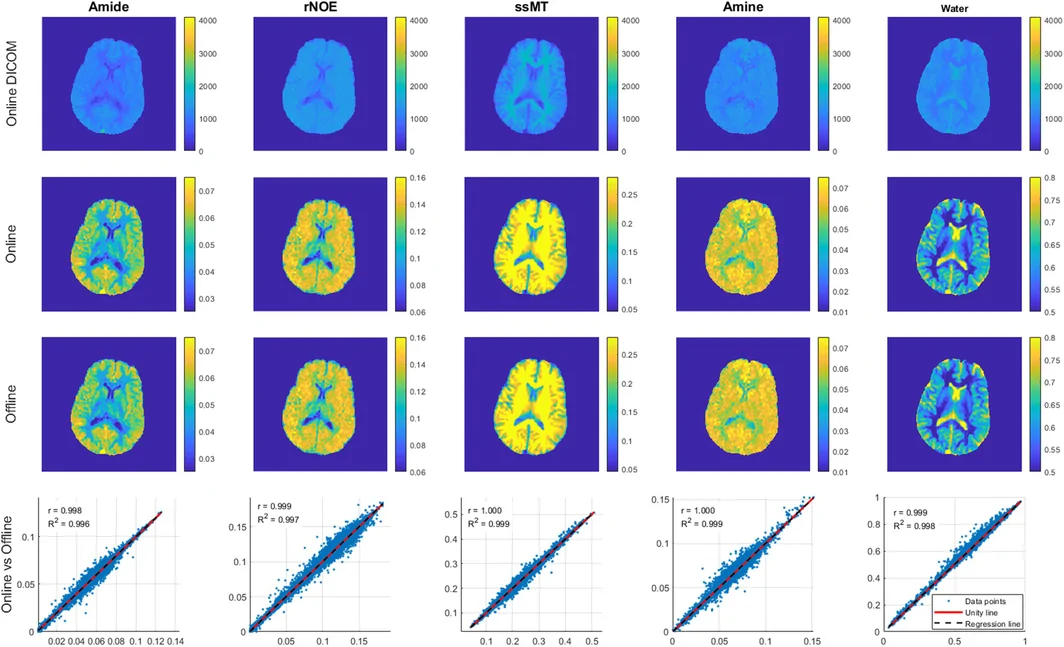
PICAE, the figure shows a comparison of a CEST evaluation. The first row shows the output of the CEST contrasts on the scanner in the typical DICOM color range between 0 and 4096. The second row shows the same maps scaled back with the corresponding values from the INI file. The third row shows the images from an offline evaluation. The same CEST offset images were used for the evaluation. The last row shows the scatter plot between the online and offline evaluation of all voxels within the 3D mask.

### 3.3 MT MRF

The results of the MT MRF implementation illustrate the steps necessary to achieve optimized results (Figure 4). The pure implementation without corrections (Figure 4, Top) shows strong inhomogeneities in the f_ss_ and k_ssw_ map. This is to be expected with B_1_ excitation fields in CP mode during UHF measurements. The center row shows that these can be mitigated with a direct use of the B_1_ map as neural network input. In the bottom row, we have artificially added realistic noise to the training data for the neural network, which leads to a further increase in performance in the k_ssw_ map (a clearer separation between the white matter and gray matter, with similar trends compared to previous literature [35]). In the context of our hybrid approach, the MRF results emphasize the importance of simplified and fast deployment of better networks.

**FIGURE 4:**
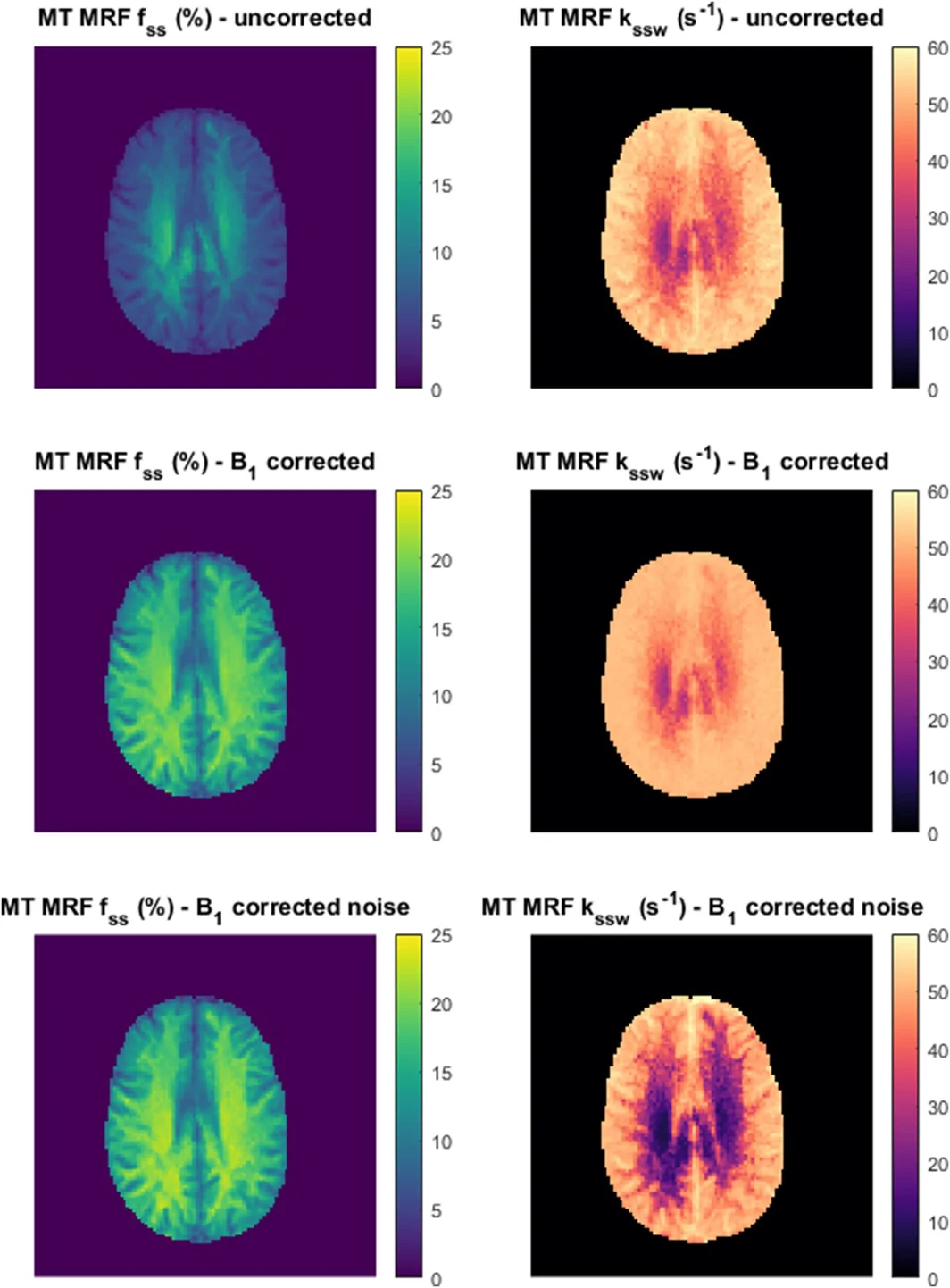
Semisolid MT MRF parameter maps. The MRF MT maps in the top row did not receive B_1_ inhomogeneity as an explicit input to the network. As a results, large differences in intensity across the same tissue (e.g., white matter) are shown. With the B_1_ correction (via explicit consideration as a network input) in the center row, these effects are reduced for the f_ss_ map. The bottom row shows results that were obtained by using the network that explicitly considered the B1 inhomogeneity per pixel, and used realistic noise injection, yielding reduced artifacts in the k_ssw_ map.

### 3.4 Readout Comparison

A key feature of the proposed framework is that the CEST evaluation pipeline remains fixed and independent of the chosen readout. To demonstrate this, we compared several readout configurations ranging from a fully sampled 2D Cartesian readout to accelerated prototype sequences with deep learning reconstruction, while keeping the CEST preparation and evaluation unchanged. Integration into a vendor prototype sequence with deep learning (DL) reconstruction presented above was also successful. As can be seen in Figure 5, both the cGRE sequence in the first column and the DL sequence with a similar acceleration in the next columns deliver nearly identical values. However, the biggest improvement in the prototype sequence is that acceleration is now possible up to 12, enabling an isotropic resolution of 1 mm whole brain. The different resolutions and acceleration methods of the readouts have an expected influence on the CEST maps. As shown in Figure 5, the high‐resolution dlGRE_C12 (E, J) in particular shows significantly more detailed images compared to the lower resolutions. The DL_C6 evaluation using CAIPIRINA 6 and NN‐based reconstruction shows increased blurring and thus performs worse than GRAPPA 6 and conventional reconstruction. The sagittal view shows that the whole brain approach benefits from the isotropic resolution of dlGRE_C12. Although the slices still appear more uneven than the transverse slices, clear improvements can be seen. The hybrid images (B, G) appear somewhat noisy. The sagittal resolution of dlGRE_C6 shows the greatest weaknesses here; unfortunately, anatomical features can only be guessed at. Noise can also be detected in the dlGRE_C12, but this can be reduced by averaging the layers to the resolution of the hybrid and dlGRE_C6. This can be seen in row (iv). The maps in the hybrid and also the dlGRE_C12, which are still noisy, appear significantly less noisy and still show finer structures such as in the gyrus.

**FIGURE 5:**
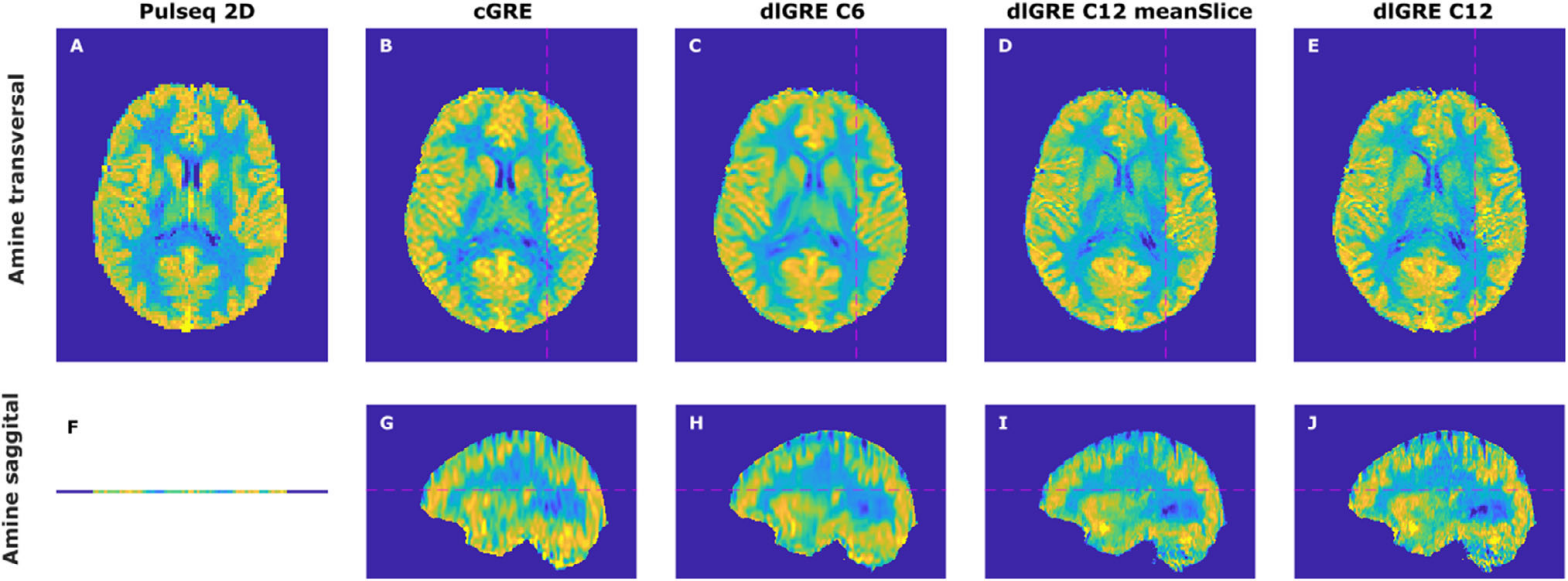
Comparison of a 2D full Pulseq solution with the conventional GRE hybrid readout (cGRE) using GRAPPA 6, the Vendor readout with Deep Learning reconstruction and CAIPIRINIA 6 (dlGRE_C6), and the DL readout with CAIPIRINIA 12 (dlGRE_C12). In order to ensure comparability in terms of layer thickness, the layer thickness of dlGRE_C12_meanSlice was subsequently adjusted to the resolution of the other two readouts. For better comparability, all CEST maps were resliced to the resolution of the dlGRE_C12. It can be seen that the hybrid has a slight difference in contrast in the edge areas compared to the WIP readouts. The dlGRE_C6 shows significantly increased blurring, especially in the Z direction. In dlGRE_C12, fine structures such as those of the gyrus can be seen more clearly, but they also appear slightly noisy. In the low‐resolution readouts, these details disappear, but so does the noise. The subsequently increased layer thickness appears less noisy than before, but still shows the increased level of detail.

One distinction from the DL closed‐source sequence is the ability to use B_0_ and B_1_ maps from the preparation scan routine. As shown in the Figure S1, this may have a slight effect on the contrast. The Supporting Information also includes an overview of all outputs from the PICAE network. To confirm that the pixel‐wise evaluation is not affected by anatomical anomalies, representative CEST maps from two patients are provided in Figure S4.

### 3.5 Reproducibility

The difference maps shown in Figure 6 and Figure S3 demonstrate the high reproducibility of the proposed method across sessions. For Subjects 1–3, the coefficient of variation (CoV) remains around 5% across all contrasts, while Subject 4 shows a moderately elevated but still acceptable CoV of approximately 10%, values consistent with the magnitude of previously reported CEST reproducibility studies [36]. The difference maps for PICAE and MRF reveal no spatially structured deviations between sessions, indicating that these contrasts are robust to day‐to‐day variability. For the B0 and B1 maps derived from the deepWASABI method, faint residual field structures are visible in regions of strong field variation, which is consistent with the known sensitivity of these maps to local susceptibility and RF inhomogeneity.

**FIGURE 6:**
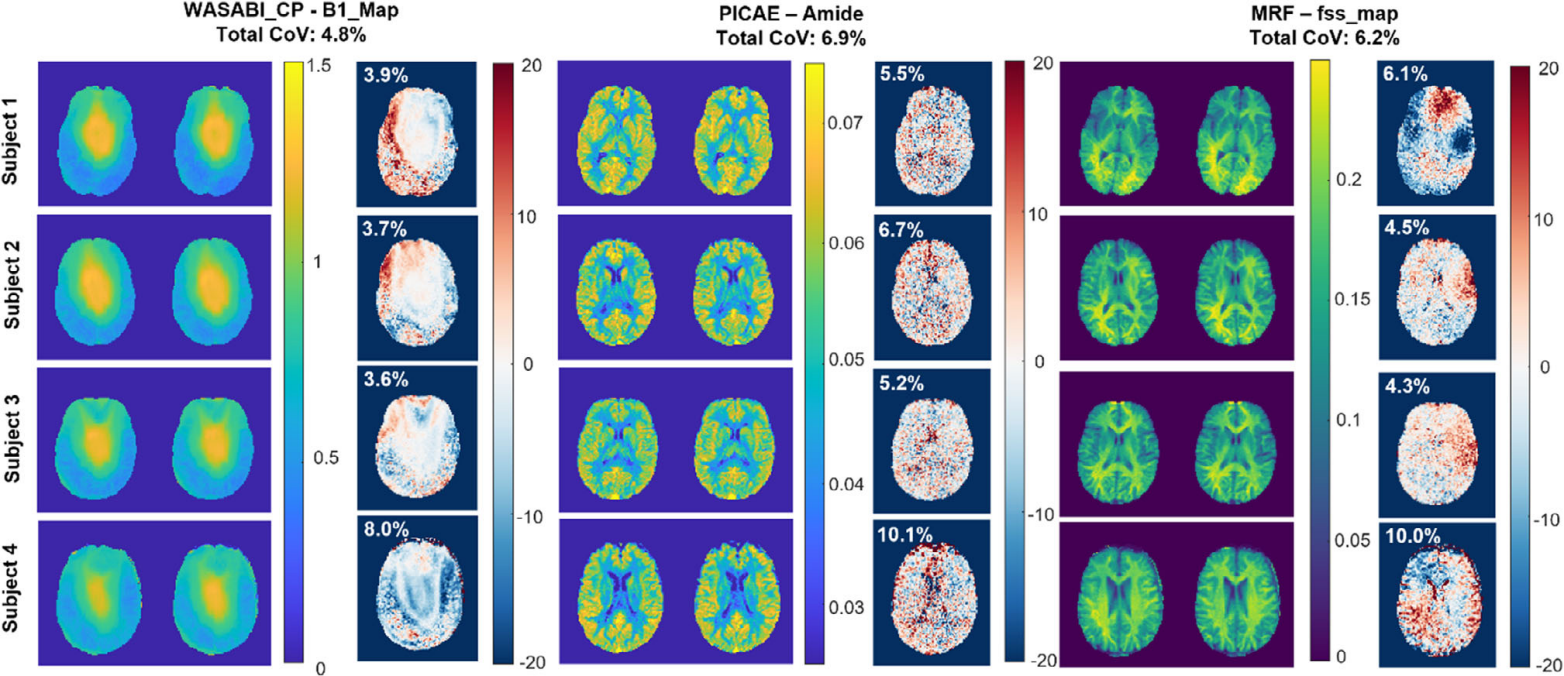
Reproducibility assessment of the three implemented methods. Representative online‐reconstructed parameter maps from two separate scanning sessions are shown side by side for deepWASABI B_1_, PICAE Amide, and MRF contrasts. A comprehensive overview of all parameter maps is provided in Figure S3. Visual inspection of the paired maps reveals no discernible differences between sessions. Voxel‐wise percentage difference maps highlight the inter‐session deviations, which remain at a low level across all contrasts. The coefficient of variation (CoV), computed over the entire masked brain volume for each individual difference map, is overlaid on the corresponding difference image and lies around 5% across subjects and contrasts. Subject 4 is an exception, with CoV values reaching approximately 10%. The overall CoV across all four subjects for each contrast is displayed below the respective parameter map title.

## 4 Discussion

In this work we presented a hybrid sequence and reconstruction framework. This approach combines a flexible, pTx‐enabled preparation block using the open‐source Pulseq standard with any open‐ or closed‐source snapshot readout, and an interchangeable ONNX‐based neural network for online post‐processing. Beyond integrating these components, we developed the ONNX inference format with online B1 correction, realized a full 2D Pulseq readout, and deliberately included a further evaluation approach the framework was not originally built around, showing that it serves as a blueprint for adoption by other groups. Together, these elements open up numerous possibilities in clinical research.

Following Herz et al. [10], our method allows the preparation methodology to be changed almost arbitrary. Existing evaluation preparations, for example, shared via Pulseq CEST [10, 27] can be loaded to this hybrid sequence and compared to the method of interest. In addition, users can take advantage of the new pulse‐specific RF shimming function. We have demonstrated that this can be used both for mode‐specific B_1_ and B_0_ mapping using WASABI [29]. For CEST preparation, we have limited ourselves to MIMOSA mode, but developers can use their optimized shim modes [37, 38], leading to more homogeneous B_1_^+^ excitation fields. As an example, the B1 maps of the PUSHUP approach [21] can be found in Figure S2. Specially developed shims can be particularly beneficial outside the head, where novel and custom‐built pTx coils can be utilized.

In addition, it is possible with the new framework to change the evaluation network in parallel. Existing evaluation methods [6, 8, 9, 39, 40, 41, 42] that are already based on neural networks can be converted to the ONNX standard, accessing an online evaluation on the scanner. An external post‐processing step by an experienced user is no longer necessary. The developer can set up once the preparation pulses together with the evaluation network on the scanner. Further measurements can be carried out by conventional MRI operators. To demonstrate that the framework can incorporate approaches beyond those it was originally built around, we integrated a semisolid MT‐MRF approach [16] and adapted it to the hybrid framework together with its originators, who joined this work as co‐authors. The network, originally designed for 3 T, was converted to the ONNX format, equipped with the B1 correction required at 7 T, and subsequently re‐optimized. The corresponding tools, such as single‐shim‐mode B_1_ mapping, are now part of the open‐source Python test framework and are available to other developers.

### 4.1 Sequence and Reconstruction Ecosystems in MRI

Sequence and reconstruction ecosystems in MRI span a spectrum from fully vendor‐native to fully open‐source. Vendor‐specific programming can leverage proprietary parallel transmit preparation as well as state‐of‐the‐art readout and reconstruction techniques, including inline deployment on the scanner. However, such workflows are often difficult to inspect, share across sites, or extend without vendor support, which can hinder rapid method development and independent validation. At the other extreme, standards such as Pulseq together with open reconstruction stacks (e.g., Gadgetron [43], YARRA [44], BART [45], MRpro [46]) maximize transparency and flexibility but often sacrifice on‐scanner execution, vendor‐optimized acquisitions, or seamless clinical archiving. Veldmann et al. [47] showed that a hybrid layout, combining an open and portable sequence description with vendor‐native readout and reconstruction, can bridge these worlds while remaining practical for sites that rely on proprietary pipelines. The present framework follows a similar approach for CEST: Pulseq‐based pTx preparation remains easy to distribute and verify, the proprietary snapshot readout preserves whole‐brain coverage and reconstruction quality, and ONNX‐based post‐processing produces parameter maps directly at the scanner.

Our approach follows this hybrid concept but differs in scope. Rather than routing raw or reconstructed images to external computing systems, as done with Gadgetron [43], Yarra [44], Fire [48]/Open Recon [49], or similar frameworks, our pipeline is fully integrated into the sequence and requires no additional IT infrastructure or permissions. In contrast to the full open‐source pipeline of Veldmann et al. [47], our framework does not aim to deploy novel reconstruction algorithms. Instead, it is designed to build on established, reusable reconstructions and use the ONNX runtime for the mapping from multiple acquired images to quantitative output maps. This has practical advantages: (i) The framework is straightforward to transfer to other vendors by reusing existing vendor reconstructions alongside ONNX, (ii) deployment is reduced to copying Pulseq and ONNX assets, and (iii) the design fits well with the growing availability of Pulseq and ONNX runtimes across scanner platforms.

A key benefit of online evaluation is the immediate availability of parameter maps at the scanner, allowing direct assessment of measurement quality and reducing the risk of repeat examinations due to delayed offline processing. Neural networks and their associated INI files are saved in a designated folder on the scanner and can be selected from a drop‐down menu in the scanner interface. Sequence parameters such as FoV, resolution, and base image reconstruction can still be adjusted directly on the scanner, provided the conditions for the snapshot approach are met [13]. The resulting parameter maps are scaled to the DICOM range via the INI file and transferred to PACS like conventional MRI images, where radiologists can apply standard windowing. To support development and validation, we also provide an offline toolbox for testing custom ONNX networks on previously acquired data. The toolbox matches the on‐scanner pipeline, meaning normalization, B_1_ mapping, and parameter map scaling behave identically, so that networks can be fully tested before scanner deployment. It also includes a function to convert online‐generated DICOM maps back to physical parameter values using the corresponding INI files.

### 4.2 Validated Experiments and Future Work

As a first approach, we trained the WASABI Fit as a neural network. The network results are close to the fit. Deviations can be attributed to purely simulated training data used for this case; a re‐training of several fitted brains is expected to further improve the NN results. However, the calculation time is the crucial point here. For the fit, the data first has to be loaded onto an external PC and then calculated for a few minutes for a single fit. In the new approach, the parameter maps are available directly at the scanner after just a few seconds. With the CP and EP WASABI maps, we also demonstrated the functionality of the RF shimming Pulseq approach. To do this, we ran a WASABI Pulseq preparation in different shim modes. The NN remained the same. As a result, we obtained fundamentally different B_1_ maps with the same identical B_0_ map. At the moment, the approach is limited to magnitude images. However, this can be extended to complex data and thus also phase information. We expect this to further improve WASABI output.

The existing PICAE network has already been successfully tested in a demonstrator by us [Rajput et al. [14]]. We have further developed this sequence and the associated reconstruction to the extent that the NN can be replaced by a wider variety of ONNX networks and CEST preparations. Any prepared 3D GRE images can be used as input data for NN in a voxel‐by‐voxel manner; any number of outputs can be generated. The repeated measurements on 4 different volunteers further demonstrate the stability of the networks, as no systematic deviations were observed. Comparison of the PICAE CoV values with those reported by Fabian et al. [36] confirms consistent results. In that study, CoV values of approximately 3%–5% for gray and white matter and around 20% for CSF were reported for PICAE evaluation. The overall CoV values of 3%–7% obtained here across the full masked brain volume and all four subjects are in good agreement with these reference values, confirming the stability of the PICAE evaluation also when performed online directly at the scanner. The CoV values for the WASABI B0/B1 and MRF‐MT contrasts fall within the same range of 3%–7%, further supporting the overall reproducibility of the proposed pipeline across all contrasts.

Because the evaluation operates on a voxel‐wise basis, the presence of anatomical anomalies introduces no additional effect on the resulting CEST maps, as illustrated in two patients in Figure S4. As an example, that the approach is not limited to CEST contrasts, we chose the MT MRF approach [16, 17, 33]. We were able to convert the approach to the ONNX format (Figure 4). Further improvements could be a higher B_1_ level or a combination with the MIMOSA approach, which would not only correct B_1_ inhomogeneities but also make the excitation pulses more homogeneous.

We demonstrated the framework at a 7 T. However, it is possible to apply these methodologies to other field strengths as well. To do so, both the pulse sequences and the ONNX must be adapted accordingly.

## 5 Conclusion

The aim of this work was to integrate flexible preparation, closed‐ and open‐source readouts and evaluation of CEST maps directly into one hybrid sequence. For preparation we chose the Pulseq format, for CEST mapping the ONNX format. To demonstrate the functionality, we successfully tested three different approaches: deepWASABI, Low power CEST, and CEST‐MRF. Deploying a new CEST method with uncompromised readout is now possible by deploying Pulseq and ONNX file at the scanner, forming an integrated sequence to use them in a clinical setting without external post‐processing. This accelerates translation of new research findings of novel methods into clinical practice.

## Funding

Funding for this work was provided by the German Federal Ministry of Research, Technology and Space, Integrated Deep Learning at 7 T (IDL@7T, 13GW0682A), the German Research Foundation (DFG RU5534 MR Biosignatures at UHF ‐ 500888779) and (DFG ‐ 559955894).

## Conflicts of Interest

Junaid R. Rajput and Patrick Liebig are employed by Siemens Healthineers. Moritz Zaiss received funding from Siemens Healthineers. The remaining authors declare no conflicts of interest.

## Supporting information


**Figure S1:** An overview of all outputs from the PICAE network. The second column, Hybrid_exB1, shows the evaluation that was performed retrospectively using the B_1_ map from the preparation scan instead of the B_1_ mapping method described above. The amides are stable across all readouts. The rNOE is only slightly elevated for DL_C12. The ssMT is elevated for the hybrid GRE readout; when the B_1_ map is replaced, this effect disappears. The water map is slightly lower for the hybrid compared to using the classic B_1_ map. The B_0_ map from the network is identical across all readouts. The B1_Mimosa map differs slightly for the hybrid and shows a somewhat lower signal.
**Figure S2:** demonstrates the flexibility of the RF shimming extension within our framework by comparing several shimming configurations. The RF shimming sets were implemented based on the PUSHUP approach, comprising a whole‐brain optimized configuration (PUSHUP‐WB) and a cerebellum‐optimized configuration (PUSHUP‐CE). For reference, the MIMOSA mode, consisting of alternating CP and EP pulses, is included as a comparison. Consistent with the findings reported in the original publication, the PUSHUP configurations show modest regional improvements over the MIMOSA shim, with the benefits being most pronounced in the anatomical regions for which the respective shim was optimized. The results overall confirm that various existing RF shimming approaches can be readily integrated into our Pulseq‐based framework.
**Figure S3:** To demonstrate the reproducibility of the proposed acquisition and reconstruction pipeline, four healthy volunteers were scanned twice on separate days using the identical protocol. The contrast maps evaluated online at the scanner were rigidly registered between sessions prior to further analysis.
**Figure S4:** Representative CEST maps from two patients with anatomically conspicuous structures, in contrast to the healthy subjects shown elsewhere in this work. The first patient (PAT1 m45, Diffuse glioma, IDH1 R132H negative, right frontoparietal extending to temporal) was measured using the cGRE readout, while the second (PAT2 f35, Astrocytoma, IDH1‐mutated, CNS WHO Grade 2, left frontal) was measured using the dlGRE_c12 readout, resulting in a higher spatial resolution for the second dataset. Both measurements were acquired and evaluated online using the PICAE method. As the evaluation in this work is performed on a pixel‐wise basis, the presence of anatomical anomalies does not introduce any additional effect on the resulting CEST maps.

## Data Availability

Pulseq CEST Library (Pulseq und ONNX) [https://github.com/kherz/pulseq-cest-library/tree/MRM26_hybrid] Online ONNX Toolbox [https://gitlab.rrze.fau.de/cest/offline_onnx_parameter_estimation/-/tree/mrm.70596] Cestineers git (.pro Files und WIP .ini) [https://github.com/cest-sources/cestineers] Open access saturation transfer MRF protocol and code [16, https://github.com/momentum-laboratory/molecular-mrf] C2P Platform
