# Supplementary material for: A Modular Hybrid Framework for CEST MRI Development and Clinical Translation Using pTx‐Pulseq Preparation With Open‐ and Closed‐Source Readouts and ONNX Mapping
Source: Magn Reson Med. 2026 Sep 24;97(1):201–12. doi: 10.1002/mrm.70596 (PMC13746068; doi:10.1002/mrm.70596)
Supplement: Supplementary file 1 [file MRM-97-201-s001.docx]

# Supporting Information:

**Supporting Information Figure S1:** An overview of all outputs from the PICAE network. The second column, Hybrid_exB1, shows the evaluation that was performed retrospectively using the B_1_ map from the preparation scan instead of the B_1_ mapping method described above. The amides are stable across all readouts. The rNOE is only slightly elevated for DL_C12. The ssMT is elevated for the hybrid GRE readout; when the B_1_ map is replaced, this effect disappears. The water map is slightly lower for the hybrid compared to using the classic B_1_ map. The B_0_ map from the network is identical across all readouts. The B1_Mimosa map differs slightly for the hybrid and shows a somewhat lower signal.

**Supporting Information Figure S2:** demonstrates the flexibility of the RF shimming extension within our framework by comparing several shimming configurations. The RF shimming sets were implemented based on the PUSHUP approach [21], comprising a whole-brain optimized configuration (PUSHUP-WB) and a cerebellum-optimized configuration (PUSHUP-CE). For reference, the MIMOSA mode, consisting of alternating CP and EP pulses, is included as a comparison. Consistent with the findings reported in the original publication [21], the PUSHUP configurations show modest regional improvements over the MIMOSA shim, with the benefits being most pronounced in the anatomical regions for which the respective shim was optimized. The results overall confirm that various existing RF shimming approaches can be readily integrated into our Pulseq-based framework.

**Supporting Information Figure S3** To demonstrate the reproducibility of the proposed acquisition and reconstruction pipeline, four healthy volunteers were scanned twice on separate days using the identical protocol. The contrast maps evaluated online at the scanner were rigidly registered between sessions prior to further analysis.

Voxel-wise difference maps were computed for each contrast using one of two metrics depending on the value distribution of the map. For strictly positive-valued maps (WASABI B1, PICAE, MRF), the symmetric percentage difference was used,

$$\Delta=\frac{(M1\boldsymbol{-}M2)}{[(\mid M1\mid\boldsymbol{+}\mid M2\mid)/2]}\boldsymbol{\times}100\%$$

Normalizing by the mean of both measurements rather than a single reference avoids numerical instability when one measurement approaches zero. Voxels where the mean magnitude fell below 1% of the contrast range were excluded from the analysis. For the zero-centered B0 field map, where a signal-normalized percentage is ill-defined near the origin, the difference was normalized by the displayed value range,

$$\Delta=\frac{(M1\boldsymbol{-}M2)}{(v\boldsymbol{max}\boldsymbol{-}v\boldsymbol{min})}\boldsymbol{\times}100\%$$

This constant denominator remains stable across the entire field of view, including where the field offset crosses zero. The two metrics are rendered with distinct colormaps to make the distinction visually apparent. All difference maps are displayed on a scale of ±20%.

To quantify spatial variability, the coefficient of variation (CoV) was computed across all voxels within the brain mask for each difference map and is reported per slice. The distribution of CoV values across all subjects and slices is summarized in the panel on the left side of each figure. For the B0 maps, the CoV was computed analogously using the range-normalized difference metric described above.

The difference maps reveal small deviations in B0 and B1 in regions where these fields are known to exhibit inherent spatial heterogeneity, such as near air-tissue interfaces. The PICAE maps show no spatially structured deviations between sessions. In the MRF maps, Subject 1 shows minor localized differences reminiscent of B0-related artifacts, which are not observed in the remaining subjects. Overall, the inter-session deviations across all contrasts and subjects range from approximately 3% to 10%, demonstrating good reproducibility of the method. A slight but consistent elevation of CoV values across all contrasts is observed in Subject 4, suggesting minor subject-specific variability, potentially related to motion or physiological differences between sessions.

**Supporting Information Figure S4.** Representative CEST maps from two patients with anatomically conspicuous structures, in contrast to the healthy subjects shown elsewhere in this work. The first patient (PAT1 m45, Diffuse glioma, IDH1 R132H negative, right frontoparietal extending to temporal) was measured using the cGRE readout, while the second (PAT2 f35, Astrocytoma, IDH1-mutated, CNS WHO grade 2, left frontal) was measured using the dlGRE_c12 readout, resulting in a higher spatial resolution for the second dataset. Both measurements were acquired and evaluated online using the PICAE method. As the evaluation in this work is performed on a pixel-wise basis, the presence of anatomical anomalies does not introduce any additional effect on the resulting CEST maps.
